# Factors influencing the timing of ovarian tissue cryopreservation in young girls

**DOI:** 10.1530/RAF-24-0032

**Published:** 2024-10-25

**Authors:** Valentina Pampanini, Lena Sahlin, Elina Holopainen, Mervi Taskinen, Mikael Koskela, Kim Vettenranta, Jaana Vettenranta, Tiina Laine, Claudia Anderson, Kirsi Jahnukainen

**Affiliations:** 1 Department of Women’s and Children’s Health, NORDFERTIL Research Lab Stockholm, Childhood Oncology Unit, Karolinska Institutet and University Hospital, Solna, Sweden; 2 Unit of Endocrinology and Diabetes, Bambino Gesù Children’s Hospital, IRCCS, Rome, Italy; 3 Department of Obstetrics and Gynecology, University of Helsinki and Helsinki University Hospital, Helsinki, Finland; 4 Division of Hematology-Oncology and Stem Cell Transplantation, University of Helsinki and Helsinki University Hospital, Helsinki, Finland; 5 New Children’s Hospital, Pediatric Research Center, University of Helsinki and Helsinki University Hospital, Helsinki, Finland

**Keywords:** children, fertility preservation, gonadotoxic treatment, ovarian tissue cryopreservation

## Abstract

The aim of this study was to identify pitfalls in ovarian tissue cryopreservation protocol from referral to surgical procedures and to analyze factors associated with chemotherapy exposure of the cryopreserved tissue and decreased ovarian function in a cohort of young girls at high risk of infertility. The study population comprised 200 girls eligible for ovarian tissue cryopreservation between 2002 and 2020 at the Children's Hospital of the University Central Hospital of Helsinki (Finland). Analyses included the evaluation of the proportion of patients who underwent ovarian tissue cryopreservation, factors associated with patient selection and timing of ovarian tissue cryopreservation, and ovarian function during long-term follow-up in relation to oncological treatments. Lack of counseling was identified as the major reason for not receiving ovarian tissue cryopreservation. A longer interval from scheduling gonadotoxic therapy to cryopreservation correlated with a higher exposure to alkylating agents of the ovarian tissue. The long-term ovarian function was mainly influenced by age at the time of gonadotoxic treatment. Current selection criteria for ovarian tissue cryopreservation should be implemented in order to stratify patients at risk of infertility and timely identify those at higher risk, especially in relation to age and pubertal stage. Efforts to increase healthcare providers’ awareness and facilitate guided timing in relation to the treatment protocols are needed to guarantee early access to ovarian tissue cryopreservation for all patients at high risk of infertility.

**Lay summary:**

Girls affected by cancer may undergo aggressive treatments to cure the disease, such as chemotherapy and radiotherapy, which can harm the ovaries. These treatments often result in the destruction of the ovaries and infertility. Nowadays, an option exists to help these girls recover their fertility after the cancer has been successfully treated. A fragment or an entire ovary can be removed by surgery and frozen before the treatments begin. When the girls are well and wish as adults to start a family, this fragment can be reimplanted back into the remaining ovary to restore fertility. This technique is called ovarian tissue cryopreservation. In this article, we report on the experience of ovarian tissue cryopreservation at the Children’s Hospital in Finland for 200 girls undergoing cancer treatment with a high risk of subsequent infertility. We analyzed the proportion of girls who had ovarian tissue cryopreservation performed among those exposed to aggressive treatments and investigated the reasons why some of them did not undergo the procedure. We also examined the time passing before the procedure was done to identify potential avoidable sources of delay. Finally, we explored how the ovaries of all the girls functioned during the time after the cancer diagnosis and looked at this in relation to their cancer treatments and ovarian tissue freezing.

## Introduction

Ovarian tissue cryopreservation (OTC) is a fertility-saving option for oncological patients subjected to gonadotoxic treatments such as chemotherapy and radiotherapy. OTC is the only option for prepubertal girls unable to undergo oocyte or embryo cryopreservation, the two established procedures routinely used to preserve fertility in adult women (Wallace *et al.* 2005, Jadoul *et al.* 2010, Wallace *et al.* 2014, Wallace *et al.* 2016, Donnez & Dolmans 2017, Oktay *et al.* 2018). Despite oocyte cryopreservation being a feasible fertility preservation approach for postpubertal patients, OTC remains the sole alternative for patients whose cancer treatment cannot be delayed and for those already exposed to chemotherapy before the indication for fertility preservation. The frozen ovarian tissue retains its functionality, and when thawed and auto-transplanted, restores ovarian function in otherwise menopausal and infertile patients (Donnez & Dolmans 2017).

Twenty-five years since the first report of a live birth following the reimplantation of harvested, mostly adult, ovarian tissue, more than 200 live births have resulted from this procedure (Donnez & Dolmans 2017, Dolmans *et al.* 2020). A few case reports have documented that ovarian tissue stored prior to puberty is capable of restoring ovarian activity and fertility once transplanted back after sexual maturation (Poirot *et al.* 2012, Ernst *et al.* 2013, Demeestere *et al.* 2015, Matthews *et al.* 2018).

Clinical guidelines have recently been published to harmonize and facilitate fertility preservation among female patients with cancer (Schuring *et al.* 2018, von Wolff *et al.* 2018, ESHRE Guideline Group on Female Fertility Preservation *et al.* 2020). The International Guideline Harmonization Group recommends ovarian tissue cryopreservation for children and young adults who will be treated with cumulative doses of alkylating agents equal or above 6000–8000 mg/m², ovarian radiotherapy, and/or hematopoietic stem cell transplantation (HSCT) (Mulder *et al.* 2021). The impact of previous chemotherapy on the quality of the harvested ovarian tissue is still a matter of debate. A recent review from five leading European centers on ovarian tissue transplantation concluded that, in adult women, previous exposure to chemotherapy does not decrease the rate of success of the procedure and is no longer regarded as a contraindication for OTC. However, a lower rate of pregnancies was found among women exposed to chemotherapy, including alkylating agents (Dolmans *et al.* 2021). In line with this observation, we have previously shown that exposure to alkylating agents at a median dose of 6100 mg/m^2^ before OTC negatively affects the ovarian follicle pool, steroid production, and stromal compartment in girls and young women (Pampanini *et al.* 2019). Alongside these considerations, the timing for OTC is influenced by several logistical aspects. Patients need to be timely identified and informed about their infertility risk and the opportunity for OTC. Fertility preservation relevance may fade in situations of emotional stress and complex management related to a cancer diagnosis (Wallace *et al.* 2016, Lampic & Wettergren 2019).

In our retrospective study, we report a cohort of consecutive (years 2002–2020) pre-pubertal and adolescent girls eligible for OTC due to a high risk of infertility. With the aim of identifying pitfalls for potential protocol optimization, we analyzed logistical and clinical factors affecting fertility counseling, selection, and timing of OTC. Analyses also included evaluation of factors associated with chemotherapy exposure of the cryopreserved tissue and decreased ovarian function after sexual maturation.

## Materials and methods

### Study population and OTC

We analyzed a cohort of 200 girls eligible for OTC between the years 2002 and 2020 at the Hospital for Children and Adolescents, Helsinki University Hospital (Finland). Patients were offered the experimental procedure of OTC if they were, based on the available evidence from the literature (Sanders *et al.* 1996, Howell & Shalet 1998, Teinturier *et al.* 1998, Thibaud *et al.* 1998, Chiarelli *et al.* 1999, Meirow 2000, Wallace *et al.* 2003), at very high risk of premature ovarian insufficiency (POI) (>80%) due to the planned treatments (allogenic/autologous HSCT or radiotherapy with the ovary in the field). The same criteria later became a part of the guidelines on fertility preservation by the Nordic Society of Paediatric Haematology and Oncology (NOPHO 2013). OTC was offered as part of a research protocol approved by the Ethics Committee of Helsinki University Hospital (Dnr 340/13/03/03/2015). A hospital license from Helsinki University Hospital was obtained to collect register data of patients with an indication for OTC (HUS 248/2019). Ovarian stimulation and oocyte retrieval were not performed, as the majority of pubertal females had undergone chemotherapy before they became eligible for fertility preservation. Embryo cryopreservation was not feasible, as the study was conducted in a pediatric unit.

We collected data on patient characteristics (i.e. age and pubertal status at OTC), diagnosis, indication for OTC, time from indication to OTC, treatment characteristics, survival time, whether information on the fertility risk and preservation counseling was provided, and the reasons for not performing OTC in those who received fertility counseling. A standardized approach was employed for recording patient journals. This included the provision of information to parents/patients regarding the research protocol and consent forms, as well as details about participation, reasons for declining participation, and the cancellation of the biopsy procedure following the receipt of information.

Indications for OTC were categorized according to the primary reason for gonadotoxic therapy, as follows: HSCT for severe hematological or immunological disease, metastatic cancer, HSCT or abdominal irradiation as part of the initial cancer treatment protocol according to stratifying factors for high risk at diagnosis (high leucocyte count, genetic subtypes or tumor histology, excluding primary metastatic cases), poor response to induction therapy, cancer relapse, and second tumor. Exposure to alkylating agents was calculated as the cyclophosphamide equivalent dose (Pitetti *et al.* 2013) as described by Green *et al.* (2014), and exposure to anthracyclines was calculated as the cumulative doxorubicin isotoxic equivalent (DIE) dose using the conversion factors 1 for doxorubicin, 0.833 for daunorubicin, 5.0 for idarubicin, and 4.0 for mitoxantrone (Shankar *et al.* 2008). The exact gonadal irradiation dose was calculated for all cases exposed to TBI (10 Gy) or whole abdominal irradiation, and in one case the exact dose to the ovaries was available from dosimetry. In all the other cases (irradiation to the retroperitoneum, kidney, spine, or tumor area), the irradiation dose to the ovaries was estimated to be equal to 50% of the local irradiation dose used. Cancer treatment exposures (CED, DIE, TBI, gonadal irradiation dose) were available only for patients who underwent OTC.

Before OTC, the parents and, when appropriate, the patients received verbal and written information about the research project and gave their written informed consent. All patients and their parents received counseling on fertility preservation from a specialist. However, information on why OTC was not performed was unavailable for some patients. Exclusion criteria were of high bleeding and/or infection risk. The inclusion and exclusion criteria remained unchanged throughout the entire study period. The surgical intervention was performed in most cases at the same time as another procedure (central venous catheter positioning or removal of abdominal mass; *n* = 59) through laparoscopy, based on the clinical necessity and innovative nature of OTC. A variable proportion (25–100%) of the ovarian cortex was removed. In 11 patients, a unilateral oophorectomy was performed because of the risk of irreversible damage to the ovary (irradiation to the ovarian field or adverse surgical outcome). Two-thirds of the tissue were cryopreserved using a slow freezing method for clinical fertility preservation, while the remaining third anonymized and cryopreserved for quality control of the ovarian tissue, including morphological analysis and *in vitro* culture, as described by Asadi Azarbaijani *et al.* (2015) and Pampanini *et al.* (2019). Two patients had postoperative intra-abdominal bleeding, with one requiring reoperation.

The follow-up program initially included semi-annual visits, transitioning to annual visits 5 years after the cessation of therapy. These visits included pubertal evaluation, menstrual history, and laboratory examinations. We retrospectively analyzed the occurrence of spontaneous puberty and menarche, as well as the need for hormone replacement therapy (HRT) during the follow-up. HRT was administered in cases of POI, defined as follicle-stimulating hormone (FSH) levels >25 IU/L measured twice, along with one of the following: absence of spontaneous puberty (lack of breast development), lack of pubertal progression, or amenorrhea (primary or secondary). Pubertal induction was considered by the age of 11 years in girls with no signs of spontaneous pubertal development and laboratory evidence of hypergonadotropic hypogonadism (Nordenstrom *et al.* 2022). Primary amenorrhea was defined as the absence of menarche by the age of 16 years. Secondary amenorrhea was defined as the cessation of menstruation for 4–6 months after menarche was established.

In order to evaluate how well the OTC group represents our inclusion criteria, we retrieved information on age and pubertal status at treatment, diagnosis, treatment characteristics, and survival time from the quality register data of the Hospital for Children and Adolescents, Helsinki University Hospital, for the 116 females who were eligible but did not undergo OTC.

### Statistical analyses

Statistical analyses were performed using the SigmaStat (v 11.00) package (SPSS), SPSS statistical software, version 25, and R version 3.6.0 (R Core Team 2019). The Mann–Whitney *U* test or Student’s *t*-test was used to compare continuous variables between two groups, after testing the normal distribution by the Shapiro–Wilk test. The Fisher’s exact test and *χ*^2^ test were used for dichotomous and polytomous categorical variables, respectively. The Kruskal–Wallis test was used to study associations between the time from OTC indication to OTC and the following: diagnosis, indication for OTC, center of origin, and Tanner stage at OTC. It was also used to analyze CED and DIE exposures before OTC in relation to different indications for OTC in malignant cases. If a significant difference occurred in the Kruskal–Wallis test, post hoc analyses were performed using the Mann–Whitney *U* test. The Spearman’s rank correlation was used to analyze the association between the time from OTC indication to OTC and the following: age at diagnosis, CED, and DIE exposures before OTC in malignant cases. It was also used to study associations between hormonal measurements, CED and DIE lifetime exposures, radiotherapy, time from sterilizing treatment, and age at sterilizing treatment. The study date was 1st December, 2019 or the date of death, whichever came first. Overall survival (OS) was defined as the time from potentially sterilizing therapy (HSCT or irradiation) to the study date and was estimated using the Kaplan–Meier method (Kaplan & Meier 1958). The cumulative incidence of HRT was illustrated using the Kaplan–Meier method, with age serving as the time scale. Differences between groups in Kaplan–Meier analyses were assessed with the log-rank test. The occurrence rate of HRT (age as time scale) was evaluated using a Cox proportional hazards model, in which explanatory variables included pubertal status at treatment (pre-pubertal or pubertal), treatment type (HSCT or irradiation), disease type (malignant or non-malignant), and whether the patient had a relapsed disease or a secondary malignancy (yes or no); the results are reported as hazard ratios (HRs) and their 95% CI.

## Results

### Description of the patient characteristics and fertility counseling

**Figure 1:**
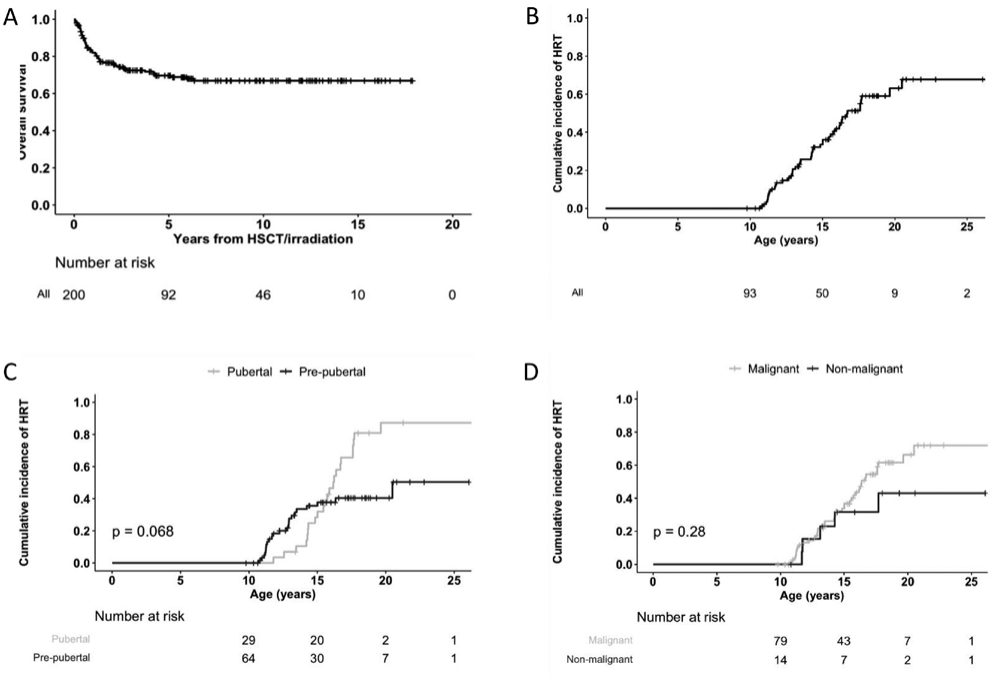
Kaplan–Meier curves representing (A) overall survival from the time of sterilizing treatment; (B) overall cumulative incidence of HRT with age as time scale; (C) cumulative incidence of HRT stratified according to pubertal status at sterilizing treatment; and (D) cumulative incidence of HRT stratified according to the type of diagnosis (non-malignant vs malignant).

In total, 200 patients were at high risk of infertility and thus eligible for OTC between 2002 and 2020 (Table 1); OS is shown in Fig. 1A. Of all patients, 121 (61%) were counseled on the risk of infertility, and 112 (56%) received verbal and written information about fertility preservation options. Of those who received information on fertility preservation, 84 (75%) successfully underwent FP (Table 2). OTC was not performed mostly because exclusion criteria were fulfilled, such as risk of bleeding or low neutrophil count. Lack of consent from the families accounted for one-third of cases who did not have OTC despite appropriate counseling. Few cases were explained by other clinical and logistic issues (Table 2).

**Table 1:** Clinical characteristics of 200 patients eligible for OTC during 2002–2020 in Helsinki Children’s Hospital. Data are presented as mean ± s.d. or as *n* (%).

Characteristics	OTC performed	OTC not performed	*P*
*n* (%)	84 (42)	116 (58)	
Follow-up, *n*	59	82	
Diagnosis			0.886^†^
Acute leukemia	27 (32.1)	41 (35.3)	
JMML, MDS, CML	5 (6)	8 (6.9)	
Solid tumors	36 (42.9)	47 (40.5)	
SAA including Fanconi anemia	10 (11.9)	9 (7.8)	
Other non-malignant diseases⁋	6 (7.1)	11 (9.5)	
Indication for OTC			0.132^†^
HSCT	77 (91.7)	105 (90.5)	
Abdominal irradiation	7 (8.3)	11 (9.5)	
Age (y) at sterilizing therapy	7.4 ± 4.7	7.1 ± 5.2	0.390^‡^
Pubertal stage at sterilizing therapy			0.551^†^
Tanner 1	66 (78.6)	91 (78.4)	
Tanner 2–3	10 (11.9)	9 (7.8)	
Tanner 4–5	8 (9.5)	16 (13.8)	
Type of HSCT			1.000^&^
Allogeneic	52 (67.5)	70 (66.7)	
Autologous	25 (32.5)	35 (33.3)	
Conditioning regimens			0.063^&^
Myeloablative with TBI	35 (45.4)	33 (31.4)	
Myeloablative without TBI	42 (54.6)	72 (68.6)	
Donor type in allo HSCT			**0.041**^&^
Related	9 (17.3)	24 (34.3)	
Non-related	43 (82.7)	46 (65.7)	
Chronic GVHD after allo HSCT			0.110^&^
Yes	10 (18.9)	6 (8.6)	
No	43 (81.1)	64 (91.4)	
Leukemia remission status			0.636^&^
CR 1	16 (57.1)	22 (51.2)	
Relapse	12 (42.9)	21 (48.8)	
FSH (IU/L)	42.5 ± 38.0	39.3 ± 48.0	
LH (IU/L)	22.6 ± 24.0	17.3 ± 21.0	
E2 (nmol/L)	0.2 ± 0.3	0.1 ± 0.2	
AMH (µg/L)	0.2 ± 0.3	1.8 ± 5.8	
AMH SDS	−1.3 ± 0.4	−0.8 ± 1.7	

^†^*χ*^2^ test; ^‡^Mann–Whitney *U* test; ^&^Fisher’s exact test; ^⁋^includes immunodeficiencies, HLH, metabolic.

AMH, anti-Müllerian hormone; CML, chronic myelogenous leukemia; CR, complete remission; E2, estradiol; FSH, follicle-stimulating hormone; GVHD, graft versus host disease; HLH, hemophagocytic lymphohistiocytosis; HSCT, hematopoietic stem cell transplantation; JMML, juvenile myelomonocytic leukemia; LH, luteinizing hormone; MDS, myelodysplastic syndrome; OTC, ovarian tissue cryopreservation; SAA, severe aplastic anemia; SDS, standard deviation score; TBI, total body irradiation.

**Table 2:** Information about fertility counseling and reasons why OTC was not performed.

	*n* (%)
Fertility risk counseling	200
Given information about risk on infertility	121 (60.5)
Not given information or not stated in the records	79 (39.5)
Fertility preservation counseling	200
Given information about fertility preservation	112 (56)
Not given information or not stated in the records	88 (44)
Fertility preservation process	112
Fertility preservation performed	84 (75)
Fertility preservation not performed due to exclusion criteria	10 (8.9)
Family not interested in FP	8 (7.1)
Logistic issues	3 (2.7)
Clinician decision*	5 (4.5)
FP not performed, reason unknown	2 (1.8)

*Re-evaluation of the opportunity to perform FP taking into account previous gonadotoxic exposure (too high) or risk of infertility (lower than initially assigned).

FP, fertility preservation; OTC, ovarian tissue cryopreservation.

### Ovarian tissue cryopreservation

Of the total of 200 patients eligible for OTC, 84 (42%) received the procedure. The percentage of OTC performed increased from 34% between 2002 and 2007 to 48% between 2014 and 2019. The most common indication for OTC was a high-risk classification at diagnosis (38%), followed by relapse (24%). The percentage of non-malignant conditions among the indications for OTC increased from 10% during the period from 2002 to 2007 to 38% between 2014 and 2020. Among the malignant conditions, the proportion of different indications for OTC was substantially unchanged over time.

Detailed clinical data according to whether OTC was performed are shown in Table 1. Patients who underwent OTC were comparable to those who did not have OTC performed in terms of age and pubertal stage at the time of sterilizing therapy, diagnosis, and indication for OTC. We performed a sub-analysis on patients who were eligible for OTC due to a planned HSCT (Table 1). No difference in the frequency of allogeneic vs autologous HSCT or leukemia remission status (CR1 vs relapse) occurred between patients with and without OTC. However, there was a tendency for a higher proportion of performed OTC among patients with TBI than in those with chemotherapy-based conditioning (*P* = 0.063), and a significantly higher proportion of OTC was performed in patients with an unrelated donor than in those with a related donor (*P* = 0.041) (Table 1).

### Timing of OTC

**Figure 2:**
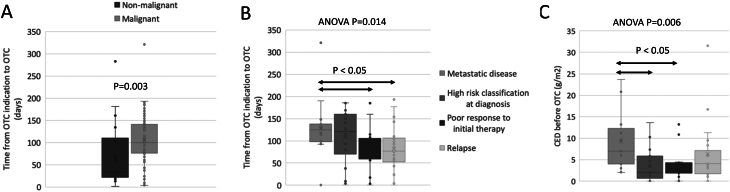
Box plot of time from OTC indication (i.e. time from the date when a patient was scheduled for high gonadotoxic therapy and the indication to OTC was established) to OTC execution according to (A) the type of diagnosis (non-malignant vs malignant); (B) the indication for OTC (primary metastatic diseases, non-metastatic cases with high-risk disease at diagnosis, non-metastatic cases with poor response to initial therapy, relapsed diseases); (C) CED exposure before OTC according to the indication for OTC.

Non-malignant diagnoses showed a shorter median time (35 days, range 3–276) from indication to OTC than the malignant ones (101 days, range: 3–314; *P* = 0.003) (Fig. 2A). OTC was performed in a shorter median time for the relapsed cases (77 days, range: 4–185 days; *P* = 0.011) and for those with a poor response to primary therapy (78 days, range: 3–181 days; *P* = 0.014) as compared to metastatic diseases (128 days, range: 91–226 days) (Fig. 2B). Tanner stage at OTC did not play a significant role in the OTC timing, which also did not significantly change between different centers of origin (data not shown). There was no statistically significant (Kruskal–Wallis test *P* = 0.13) secular change in the median time from decision to procedure of OTC (104 days (IQR: 67–160), 95 days (IQR: 74–138), and 71 days (IQR: 41–112), respectively) or in the proportion of malignant diseases as a reason for OTC (81% in 2002–2007, 79% in 2008–2013, and 78% in 2014–2019) across the diagnostic eras of 2002–2007, 2008–2013, and 2014–2019.

Patients with metastatic cancer had the highest median CED exposure (7.0 g/m^2^, range: 2.0–21.7) before OTC compared to cases with a poor response to primary cancer therapy (2.2 g/m^2^, range: 0–13.2; *P* = 0.003) and those classified as high risk at primary cancer diagnosis (2.0 g/m^2^, range: 0–13.7; *P* = 0.001) (Fig. 2C). Spearman’s correlation analysis showed a significant correlation between higher CED exposure before OTC and a longer time to OTC (*ρ* = 0.337, *P* = 0.005). Median DIE exposure before OTC was highest among patients with relapsed (180 mg/m^2^, range: 0–430) and metastatic cancers (150 mg/m^2^, range: 0–360).

### Ovarian function after high-risk gonadotoxic treatment

Altogether, 59/84 (70%) patients who underwent OTC and 82/116 (71%) who did not, became long-term survivors and attended a follow-up program at an outpatient clinic. Pubertal development and the need of HRT were analyzed in this sub-cohort. The natural history of ovarian function in the whole cohort is shown in Supplemental Figure 1 (see section on supplementary materials given at the end of this article). Follow-up data of the ovarian function before and after sterilizing treatment were available for 94 survivors (38 OTC, 56 no OTC). Of the 94 patients, 46 (49%) received HRT during the follow-up period; the cumulative incidence of HRT was 63% (95% CI: 47–74%) at age 20 years (Fig. 1B). Twenty-three of 66 (35%) patients who were pre-pubertal at the time of the sterilizing treatment were treated with HRT during follow-up. Of the 28 patients who were pubertal at the time of the sterilizing treatment, 23 (82%) needed HRT during follow-up. The cumulative incidence of HRT was higher among patients who were already pubertal at the time of OTC (compared to pre-pubertal ones) and in malignant cases (compared to non-malignant ones), but the differences did not reach statistical significance (Fig. 1C and D). In the Cox proportional hazards model, the occurrence rate of HRT was significantly higher in pubertal patients (HR: 2.01, 95% CI: 1.10–3.68; *P* = 0.022), whereas treatment with HSCT (vs abdominal radiation) approached significance (HR: 2.82, 95% CI: 0.93–8.48; *P* = 0.066). No significant associations were observed with disease type and whether the patient had relapsed disease or a secondary cancer.

## Discussion

This study describes a large cohort of 200 consecutive pediatric and adolescent patients eligible for OTC prior to gonadotoxic treatment associated with a very high risk of infertility (risk of POI >80%) according to the NOPHO criteria used in the Nordic Countries. Altogether, 56% were counseled on fertility preservation options, and 75% of them agreed to join the research program and received OTC. This highlights the relevance perceived by patients and their families about the chance to preserve fertility. Lack of counseling accounted for the largest part of cases who did not receive OTC despite the risk of infertility exceeding 80%. Exclusion criteria and lack of consent from patients and families were identified as the major causes for not having OTC after appropriate counseling.

No differences regarding the diagnosis of malignancy, age of the patient, expected survival rate, or the referring center occurred between patients who received OTC and those who did not, suggesting that reasons for not performing OTC were not related to these factors. In patients who were eligible for OTC due to a planned HSCT, a TBI-based conditioning regimen was associated with a higher frequency of performed OTC than conditioning without TBI. This could be due to the perception of a higher risk of gonadotoxicity. Among patients treated with allogenic HSCT, OTC was more frequently performed if the donor type was unrelated than if it was related. Despite the identification of matched unrelated donors becoming considerably more likely, it still require a longer time compared to related family donors. The longer time frame to perform HSCT could have facilitated setting up the OTC procedure in these cases.

According to previous reports, a lower exposure to alkylating agents may ensure a better quality of the harvested ovarian tissue (Wallace *et al.* 2014, Pampanini *et al.* 2019). Clinical factors, such as the need to stabilize clinical conditions or the attempt to combine OTC with another procedure, may delay the harvest. During this time, the first cycles of chemotherapy are usually administered. In our cancer patients, we observed that a longer time to OTC correlated with a higher exposure to alkylating agents before OTC. The waiting time for OTC was significantly longer for patients with metastatic disease than those with relapse. Out of the 20 relapsed cases, 17 had a hematological malignancy. These are easily identified to be at risk of infertility already at the day of relapse, which may have expedited the decision to perform OTC. Metastatic cancers were mostly neuroblastoma or Ewing sarcoma, and OTC was often postponed to the time of a complete response or primary surgical operation. This observation suggests that the attempt to combine OTC with tumor operation may not be optimal to avoid exposure to chemotherapy. All efforts should be directed at shortening the time to OTC procedure when the indication is established in order to limit the toxic exposure of the ovary and reduce the damage to the cryopreserved ovarian material.

This study suggests that patients already pubertal at the time of treatment have a higher risk of ovarian failure and need for HRT when compared to prepubertal patients. This reflects the physiological decline in ovarian reserve with age, characterized by a peak in PF loss at around 14 years of age, making older patients more susceptible to POI (De Bruin *et al.* 2008, Wallace & Kelsey 2010, Thomas-Teinturier *et al.* 2013). As progressively smaller doses are required to produce ovarian failure at an older age, therapies other than HSCT or ovarian irradiation may cause a very high risk of POI in adolescent girls. In addition to a proper assessment of the gonadotoxic effects of the planned therapies, the age-related risk of POI must be taken into account when counseling patients for OTC.

OTC should be performed in a timely manner to minimize chemotherapy exposure before ovarian tissue harvesting.

This study has some limitations. First, although the total study cohort was relatively large, the variation in treatment exposures did not allow for subgroup analyses of the POI cumulative incidence in relation to different categories of treatment. With a longer follow-up, some patients might display a temporary recovery of ovarian function while others will develop ovarian insufficiency later in life. Other limitations include the retrospective nature of the data, with the lack of pre-planned data entry potentially resulting in gaps in the information needed for the analysis. The reasons for not performing OTC on some of the eligible patients were not retrievable; hence, our conclusions in this sense remain speculative.

In conclusion, our observations support the validity of the current selection criteria for OTC, with an emphasis on post-pubertal patients who are at higher risk of ovarian insufficiency with equally gonadotoxic treatment. Adaptation to adult fertility preservation guidelines should therefore be considered when the girl has reached the maturity of young adulthood. Altogether, 56% of patients who were at risk of infertility were counseled and 75% of them received OTC. Over half of the eligible patients in the total cohort did not receive OTC, for reasons that could be related to logistical factors or to the perception, experience, and knowledge of the healthcare providers. Efforts to shorten the time from indication to procedure are needed to limit the exposure to alkylating agents before OTC. We suggest that the optimal slot for fertility preservation should be pointed out in all future cancer therapy protocols to harmonize the service and increase healthcare providers‘ awareness.

## Supplementary Materials

Supplemental Figure 1

## Declaration of interest

The authors declare that there is no conflict of interest that could be perceived as prejudicing the impartiality of the research reported.

## Funding

This study was supported by grants from the Swedish Childhood Cancer Foundation (KP2020-0021; PR2017-0037) (KJ), the Finnish Cancer Society (KJ), the Finnish Foundation for Pediatric Research (KJ), as well as the Birgitta and Carl-Axel Rydbeck’s Research Grant for Paediatric Research (2020-00335; 2021-00079) (KJ). This work was also supported by the Italian Ministry of Healthhttp://dx.doi.org/10.13039/100009647 with ‘Current Research funds’.

## Author contribution statement

KJ: experimental design, data collection, interpretation of data, drafting the manuscript. VP: experimental design, interpretation of data, drafting the manuscript. MK: analyses and interpretation of data, drafting the manuscript. JV: data collection. EH, MT, KV, TL: supervision of clinical patient care. All authors made significant intellectual contributions in reviewing the manuscript and approved the final article.
