# Supplementary figures and images for: Factors influencing the timing of ovarian tissue cryopreservation in young girls
Source: Reprod Fertil. 2024 Oct 25;5(4):e240032. doi: 10.1530/RAF-24-0032 (PMC11558924; doi:10.1530/RAF-24-0032)

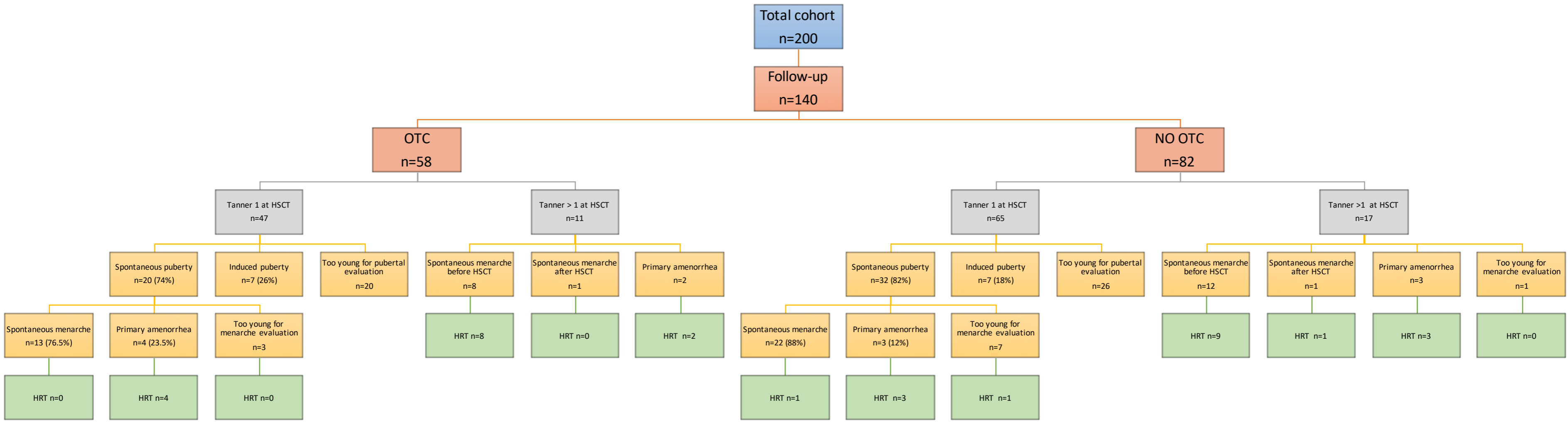

Supplement: Supplemental Figure 1 [file supplementary_figure_1.pdf]
